# Author Correction: CRISPR screens reveal convergent targeting strategies against evolutionarily distinct chemoresistance in cancer

**DOI:** 10.1038/s41467-025-56972-x

**Published:** 2025-02-12

**Authors:** Chunge Zhong, Wen-Jie Jiang, Yingjia Yao, Zexu Li, You Li, Shengnan Wang, Xiaofeng Wang, Wenjuan Zhu, Siqi Wu, Jing Wang, Shuangshuang Fan, Shixin Ma, Yeshu Liu, Han Zhang, Wenchang Zhao, Lu Zhao, Yi Feng, Zihan Li, Ruifang Guo, Li Yu, Fengyun Pei, Jun Hu, Xingzhi Feng, Zihuan Yang, Zhengjia Yang, Xueying Yang, Yue Hou, Danni Zhang, Dake Xu, Ren Sheng, Yihao Li, Lijun Liu, Hua-Jun Wu, Jun Huang, Teng Fei

**Affiliations:** 1 Key Laboratory of Bioresource Research and Development of Liaoning Province, College of Life and Health Sciences, Northeastern University, Shenyang, 110819 China; 2 National Frontiers Science Center for Industrial Intelligence and Systems Optimization, Northeastern University, Shenyang, 110819 China; 3 Key Laboratory of Data Analytics and Optimization for Smart Industry (Northeastern University), Ministry of Education, Shenyang, 110819 China; 4 Foshan Graduate School of Innovation, Northeastern University, Foshan, 528311 China; 5 Peking University Third Hospital, Beijing, 100191 China; 6 Department of Colorectal Surgery, the Sixth Affiliated Hospital, Sun Yat-sen University, Guangzhou, China; 7 Clinical Research Center, the Sixth Affiliated Hospital, Sun Yat-sen University, Guangzhou, China; 8 Guangdong Provincial Key Laboratory of Colorectal and Pelvic Floor Diseases, the Sixth Affiliated Hospital, Sun Yat-sen University, Guangzhou, China; 9 Guangdong Institute of Gastroenterology, Guangzhou, China; 10 Department of Cardiothoracic Surgery, Jinqiu Hospital of Liaoning Province, Shenyang, China; 11 Department of Thoracic Surgery, The Fourth Affiliated Hospital of China Medical University, Shenyang, China; 12 Key Laboratory for Anisotropy and Texture of Materials (Ministry of Education), School of Materials Science and Engineering, Northeastern University, Shenyang, China; 13 BeiGene Institute, BeiGene (Shanghai) Research & Development Co., Ltd, 200131 Shanghai, China; 14 Key laboratory of Carcinogenesis and Translational Research (Ministry of Education/Beijing), Peking University Cancer Hospital & Institute, Beijing, 100142 China; 15 Department of Biomedical Informatics, School of Basic Medical Sciences, Peking University Health Science Center, Beijing, 100191 China; 16 Center for Precision Medicine Multi-Omics Research, Institute of Advanced Clinical Medicine, Peking University, Beijing, 100191 China

**Keywords:** Cancer genomics, Cancer genetics, Mechanism of action

Correction to: *Nature Communications* 10.1038/s41467-024-49673-4, published online 29 June 2024

In the version of the article initially published, in the section “A systematic view on genetic maps of chemoresistance”, the sentence “Microtubule-related genes such as *KIF1C…*” has been corrected to “Microtubule-related genes such as *KIFC1…*” in the HTML and PDF versions of the article. In Supplementary Data 6, two oligo sequences were incorrectly written as ‘sgRNA_*MMACHC*-2_Forward: CACCGGGTGTATGCACACACCTGA’ and ‘sgRNA_*MMACHC*-2_Reverse: AAACTCAGGTGTGTGCATACACCC’. The correct version should be ‘sgRNA_*MMACHC*-2_Forward: CACCGCTAACACGGCCCAGATGGT’ and ‘sgRNA_*MMACHC*-2_Reverse: AAACACCATCTGGGCCGTGTTAGC’. A corrected version of Supplementary Data 6 is now available online.

## Supplementary information

Updated Dataset 6

