# Supplementary material for: CRISPR screens reveal convergent targeting strategies against evolutionarily distinct chemoresistance in cancer
Source: Nat Commun. 2024 Jun 29;15:5502. doi: 10.1038/s41467-024-49673-4 (PMC11217446; doi:10.1038/s41467-024-49673-4)
Supplement: Supplementary file 8 — Reporting Summary [file 41467_2024_49673_MOESM8_ESM.pdf]

nature portfolio

Corresponding author(s): Teng Fei
Jun Huang
Hua-Jun Wu

Last updated by author(s): Jun 2, 2024

Reporting Summary

Nature Portfolio wishes to improve the reproducibility of the work that we publish. This form provides structure for consistency and transparency in reporting. For further information on Nature Portfolio policies, see our Editorial Policies and the Editorial Policy Checklist.

Statistics

For all statistical analyses, confirm that the following items are present in the figure legend, table legend, main text, or Methods section.

n/a	Confirmed	
☐	☒	The exact sample size (*n*) for each experimental group/condition, given as a discrete number and unit of measurement
☐	☒	A statement on whether measurements were taken from distinct samples or whether the same sample was measured repeatedly
☐	☒	The statistical test(s) used AND whether they are one- or two-sided<br>*Only common tests should be described solely by name; describe more complex techniques in the Methods section.*
☒	☐	A description of all covariates tested
☐	☒	A description of any assumptions or corrections, such as tests of normality and adjustment for multiple comparisons
☐	☒	A full description of the statistical parameters including central tendency (e.g. means) or other basic estimates (e.g. regression coefficient) AND variation (e.g. standard deviation) or associated estimates of uncertainty (e.g. confidence intervals)
☐	☒	For null hypothesis testing, the test statistic (e.g. *F*, *t*, *r*) with confidence intervals, effect sizes, degrees of freedom and *P* value noted<br>*Give P values as exact values whenever suitable.*
☒	☐	For Bayesian analysis, information on the choice of priors and Markov chain Monte Carlo settings
☒	☐	For hierarchical and complex designs, identification of the appropriate level for tests and full reporting of outcomes
☐	☒	Estimates of effect sizes (e.g. Cohen's *d*, Pearson's *r*), indicating how they were calculated

Our web collection on statistics for biologists contains articles on many of the points above.

Software and code

Policy information about availability of computer code

Data collection	Raw sequencing data were generated on Illumina PE150 sequencing platform (Novogene, China). Data were also collected from public sources such as TumorPortal (http://www.tumorportal.org/), the Cancer Cell Line Encyclopedia (CCLE) (https://sites.broadinstitute.org/ccle/), NCBI Gene Expression Omnibus (GEO) database, and GEPIA (Gene Expression Profiling Interactive Analysis) (http://gepia.cancer-pku.cn/) .
Data analysis	The following softwares or algorithms were employed for data analysis: Prism 9, Flow Jo_v10, R Base 4.0.4, Circlize 0.4.13, ComplexHeatmap 2.7.11, clusterProfiler 3.18.1, dbplyr 2.1.1, dplyr 1.0.8, DESeq2 1.30.1, ggplot2 3.3.5, ggtext 0.1.1, ggvenn 0.1.9, GSVA 1.38.2, MAGeCK 0.5.9.5, MAGeCKFlute 1.10.0, UpSetR 1.4.0, tidyverse 1.3.1, enrichplot 1.10.2, and Cytoscape 3.9.1.

For manuscripts utilizing custom algorithms or software that are central to the research but not yet described in published literature, software must be made available to editors and reviewers. We strongly encourage code deposition in a community repository (e.g. GitHub). See the Nature Portfolio guidelines for submitting code & software for further information.

Data

Policy information about availability of data

All manuscripts must include a data availability statement. This statement should provide the following information, where applicable:

- Accession codes, unique identifiers, or web links for publicly available datasets
- A description of any restrictions on data availability
- For clinical datasets or third party data, please ensure that the statement adheres to our policy

The raw sequencing data generated in this study have been deposited and are publicly available in the Genome Sequence Archive in National Genomics Data

Center, China National Center for Bioinformation / Beijing Institute of Genomics, Chinese Academy of Sciences, under accession code (GSA-Human: HRA002646) at https://ngdc.cncb.ac.cn/gsa-human. Tumor suppressor genes (TSGs) and oncogenes were sourced from the COSMIC Cancer Gene Census (https://cancer.sanger.ac.uk/census/), while essential genes were derived from a previous study, and mutational profiles of the cell lines were obtained from the Cancer Cell Line Encyclopedia (CCLE, https://sites.broadinstitute.org/ccle/). Significantly mutated genes from TCGA were identified using MutSig v2.0 analysis from the GDAC data portal, focusing on genes with FDR < 0.01. The overlapping genes between these significantly mutated genes and chemoresistance genes were visualized using OncoPrint in cBioPortal (https://www.cbioportal.org/), which also provided the mutational profile of PLK4. Additionally, RNA-seq data for colorectal cancer were collected from the NCBI GEO database, including 54 samples from 18 CRC patients (normal colon, primary CRC, liver metastasis) under accession number GSE50760 (https://www.ncbi.nlm.nih.gov/geo/query/acc.cgi?acc=GSE50760), and 60 samples (30 normal colon, 30 colon cancer) under accession number GSE74602 (https://www.ncbi.nlm.nih.gov/geo/query/acc.cgi?acc=GSE74602). Source data are provided with this paper. The remaining data are available within the Article, Supplementary Information or Source Data file.

Research involving human participants, their data, or biological material

Policy information about studies with human participants or human data. See also policy information about sex, gender (identity/presentation), and sexual orientation and race, ethnicity and racism.

Reporting on sex and gender	Sex and gender were not considered in this study.
Reporting on race, ethnicity, or other socially relevant groupings	Reporting on race, ethnicity, or other socially relevant groupings were not considered in this study.
Population characteristics	Tumor tissue samples were acquired from colorectal cancer patients during surgery at The Sixth Affiliated Hospital of Sun Yat-sen University in China. Informed consent and ethical approval were obtained for this study.
Recruitment	Tumor tissues were obtained from colorectal cancer patients during surgery with informed consent. Adjacent normal tissues were obtained from resected colorectal segments with tumors.
Ethics oversight	Ethical approval was obtained from the ethical committee of The Sixth Affiliated Hospital of Sun Yat-sen University of China (2021ZSLYEC-466).

Note that full information on the approval of the study protocol must also be provided in the manuscript.

Field-specific reporting

Please select the one below that is the best fit for your research. If you are not sure, read the appropriate sections before making your selection.

[x] Life sciences [ ] Behavioural & social sciences [ ] Ecological, evolutionary & environmental sciences

For a reference copy of the document with all sections, see nature.com/documents/nr-reporting-summary-flat.pdf

Life sciences study design

All studies must disclose on these points even when the disclosure is negative.

Sample size	No statistical methods were used to predetermine the sample size. To proceed with statistical analysis, more than three samples or repeats were performed.
Data exclusions	No data were excluded from the analyses.
Replication	At least two or three independent replicates were performed in general to ensure reproducibility, and all attempts at reproducibility were successful. The number of replicates for corresponding experiments was also indicated in Methods and Figure legends.
Randomization	The experiments were not randomized.
Blinding	The Investigators were not blinded to allocation during experiments and outcome assessment.

Reporting for specific materials, systems and methods

We require information from authors about some types of materials, experimental systems and methods used in many studies. Here, indicate whether each material, system or method listed is relevant to your study. If you are not sure if a list item applies to your research, read the appropriate section before selecting a response.

Materials & experimental systems

n/a	Involved in the study
☐	☒ Antibodies
☐	☒ Eukaryotic cell lines
☒	☐ Palaeontology and archaeology
☐	☒ Animals and other organisms
☒	☐ Clinical data
☒	☐ Dual use research of concern
☒	☐ Plants

Methods

n/a	Involved in the study
☒	☐ ChIP-seq
☐	☒ Flow cytometry
☒	☐ MRI-based neuroimaging

Antibodies

Antibodies used

p53 (1:1000, Santa Cruz Biotechnology, Cat# sc-126, https://www.scbt.com/zh/p/p53-antibody-do-1)
GAPDH (1:3000, Santa Cruz Biotechnology, Cat# sc-25778, https://www.scbt.com/zh/p/gapdh-antibody-fl-335)
PLK4 (1:1000, Cell Signaling Technology, Cat# 71033, https://www.cellsignal.cn/products/primary-antibodies/plk4-e6a7r-rabbit-mab/71033)
PLK1 (1:1000, Proteintech, Cat# 10305-1-AP, https://www.ptgcn.com/products/PLK1-Antibody-10305-1-AP.htm)
BrdU (1:200, Cell Signaling Technology, Cat# 5292, https://www.cellsignal.cn/products/primary-antibodies/brdu-bu20a-mouse-mab/5292)
α-Tubulin (1:100, Proteintech, Cat# 66031-1-Ig, https://www.ptgcn.com/products/tubulin-Alpha-Antibody-66031-1-Ig.htm)
γ-tubulin (1:200, Proteintech # 15176-1-AP, https://www.ptgcn.com/products/TUBG1-Antibody-15176-1-AP.htm)
p-AKT (Thr308) (1:1000, Cell Signaling Technology, Cat# C31E5E, https://www.cellsignal.cn/products/primary-antibodies/phospho-akt-thr308-c31e5e-rabbit-mab/2965)
AKT (1:1000, Proteintech, Cat# 10176-2-AP, https://www.ptgcn.com/products/AKT-Antibody-10176-2-AP.htm)
p-GSK3β (1:1000, Ser 9) (Beyotime, Cat# AF1531, https://www.beyotime.com/product/AF1531.htm)
GSK3β (1:1000, Proteintech, Cat# 22104-1-AP, https://www.ptgcn.com/products/GSK3B-Antibody-22104-1-AP.htm)
p-ERK1/2 (1:1000, Cell Signaling Technology, Cat# 4370S, https://www.cellsignal.cn/products/primary-antibodies/phospho-p44-42-mapk-erk1-2-thr202-tyr204-d13-14-4e-xp-rabbit-mab/4370)
ERK1/2 (1:1000, Cell Signaling Technology, Cat# 137F5, https://www.cellsignal.cn/products/primary-antibodies/p44-42-mapk-erk1-2-137f5-rabbit-mab/4695)
Goat anti-Rabbit IgG (1:5000, Thermo Fisher Scientific, Cat# 31460, https://www.thermofisher.cn/cn/zh/antibody/product/Goat-anti-Rabbit-IgG-H-L-Secondary-Antibody-Polyclonal/31460)
Rabbit anti-Mouse IgG (1:5000, Thermo Fisher Scientific, Cat# 31450, https://www.thermofisher.cn/cn/zh/antibody/product/Rabbit-anti-Mouse-IgG-H-L-Secondary-Antibody-Polyclonal/31450)
Goat anti-Mouse IgG (1:500, Thermo Fisher Scientific, Cat# A-11001, https://www.thermofisher.cn/cn/zh/antibody/product/Goat-anti-Mouse-IgG-H-L-Cross-Adsorbed-Secondary-Antibody-Polyclonal/A-11001)
p21 (1:1000, Proteintech, Cat# 10355-1-AP, https://www.ptgcn.com/products/P21-Antibody-10355-1-AP.htm)
CyclinD1 (1:1000, Santa Cruz Biotechnology, Cat# sc-718, https://www.scbt.com/zh/p/cyclin-d1-antibody-m-20)
CyclinE (1:1000, Santa Cruz Biotechnology, Cat# sc-481, https://www.scbt.com/zh/p/cyclin-e-antibody-m-20)
p-Rb (1:1000, Cell Signaling Technology, Cat# 9308, https://www.cellsignal.cn/products/primary-antibodies/phospho-rb-ser807-811-antibody/9308)
Rb (1:2000, Cell Signaling Technology, Cat# 9309, https://www.cellsignal.cn/products/primary-antibodies/rb-4h1-mouse-mab/9309)
Cyclin B1 (1:1000, Proteintech, Cat# 55004-1-AP, https://www.ptgcn.com/products/CCNB1-Antibody-55004-1-AP.htm)
CDK1 (1:2000, Proteintech, Cat# 19532-1-AP, https://www.ptgcn.com/products/CDC2-Specific-Antibody-19532-1-AP.htm)

Validation

All antibodies are commercially available and have been validated by vendors as well as previously published studies.

Eukaryotic cell lines

Policy information about cell lines and Sex and Gender in Research

Cell line source(s)

All cell lines used in this study were obtained from American Type Culture Collection (ATCC).

Authentication

Authentication was not performed after obtaining these cells.

Mycoplasma contamination

All cell lines tested negative for mycoplasma contamination.

Commonly misidentified lines
(See ICLAC register)

No commonly misidentified cell lines were used.

Animals and other research organisms

Policy information about studies involving animals; ARRIVE guidelines recommended for reporting animal research, and Sex and Gender in Research

Laboratory animals

All animal experimental procedures were performed according to the Guidelines for the Care and Use of Laboratory Animals and

	were approved by the Biological and Medical Ethics Committee of Northeastern University (#NEU-EC-2021A020S). Around 6-week-old SFP-BALB/cA-nu female mice were purchased from Beijing HFK Bioscience Co.,Ltd. (Beijing, China). All mice were fed in standard individual ventilated cages, and maintained with 12h : 12h light cycle, 24-26°C room temperature and 40%–60% relative humidity.
Wild animals	No wild animals were used in the study.
Reporting on sex	All the mice were male in this study.
Field-collected samples	This study did not involve samples collected from the field.
Ethics oversight	All animal experimental procedures were performed according to the Guidelines for the Care and Use of Laboratory Animals and were approved by the Biological and Medical Ethics Committee of Northeastern University (#NEU-EC-2021A020S).

Note that full information on the approval of the study protocol must also be provided in the manuscript.

Flow Cytometry

Plots

Confirm that:

- [ ] The axis labels state the marker and fluorochrome used (e.g. CD4-FITC).
- [ ] The axis scales are clearly visible. Include numbers along axes only for bottom left plot of group (a 'group' is an analysis of identical markers).
- [ ] All plots are contour plots with outliers or pseudocolor plots.
- [ ] A numerical value for number of cells or percentage (with statistics) is provided.

Methodology

Sample preparation	Cell cycle analysis. For cell cycle assays, two staining methods (BrdU + PI or PI only) were used. For BrdU plus propidium iodide (PI) staining, 75 μM BrdU was added to the medium one hour before harvesting the treated cells. Cells were fully digested and washed with PBS. Add 90% ethanol dropwise while vortexing to fix the cells. Protect from light at 4°C overnight. Resuspend and wash samples with PBS. Add 0.5 mL of 2 M HCl (0.5% Triton X-100) dropwise while vortexing and incubate for 30 min at room temperature followed by washing with PBS. Add 1 mL of 100 mM sodium borate solution (pH = 8.5), resuspend the cells, and centrifuge at 700 x g for 5 min. Discard the supernatant, add 1 mL of 3% BSA blocking solution prepared in PBST, and incubate at room temperature for 30 min followed by centrifugation to remove the supernatant. Add 100 μL BrdU primary antibody (Cell Signaling Technology # 5292; 1:200 dilution in PBST with 1% BSA) and incubate for one hour at room temperature. After washing with PBST, add 100 μL mouse fluorescence secondary antibody (Thermo Fisher Scientific # A-11001; 1:500 dilution in PBST with 1% BSA). Protect from light and incubate at room temperature for 30 min followed by washing with PBST. Add 350 μL PI/RNase Staining Buffer (BD # 550825) and incubate at room temperature for 30 min while protecting from light. Samples were then filtered through 200-mesh nylon membrane and cell cycle status was analyzed by running on a flow cytometer (BD LSRFortessa). For PI staining, cells were collected and washed once with PBS. Fix cells with 70% pre-cooled ethanol at -20°C overnight. After washing with PBS, add 500 μL PI/RNase Staining Buffer and incubate at room temperature for 30 min in the dark. Use 200-mesh nylon membrane to filter the samples and proceed to flow cytometry for cell cycle analysis. The gating strategy for flow cytometry analysis was shown in Fig. S19.<br><br>Apoptosis assay. For apoptosis assays, cells were stained by two methods (PI + Annexin V or PI + Hoechst) and analyzed by flow cytometry. For PI plus Annexin V (FITC) staining, cells were collected and washed once with PBS. Take 1×105 resuspended cells, add 195 μL Annexin V-FITC binding solution, 5 μL Annexin V-FITC (Beyotime # C1062) and 10 μL of PI staining solution (Beyotime # C1062). Mix gently and incubate in the dark for 10-20 min at room temperature. Cells were passed through 200-mesh nylon membrane and analyzed on a flow cytometer (BD LSRFortessa). For PI plus Hoechst staining, about 1 million cells were collected for each cell sample in a 1.5 mL centrifuge tube. The cell pellet was resuspended in 0.8 mL of cell staining buffer. Add 5 μL Hoechst staining solution (Beyotime # C1056) and 5 μL PI staining solution. Mix well and incubate on ice or 4ºC for 20-30 min. Pass cells through 200-mesh nylon membrane and proceed to flow cytometry analysis. The gating strategy for flow cytometry analysis was shown in Fig. S19.
Instrument	flow cytometer (BD LSRFortessa)
Software	FlowJo
Cell population abundance	A minimum of 10,000 events were aquired per sample following debris and doublet exclusion.
Gating strategy	Cells were gated on SSC-Area and FSC-Area for morphology to remove debris. Doublets and cell aggregates were excluded by gating in single cells (PI-Width vs. PI-Area). The cell population gated in after debris and doublet exclusion was then used to create single-staining histograms (cell cycle based on PI), DNA content analysis (based on PI) and double-staining quadrants (BrdU and PI for cell cycle analysis).<br>Cells were gated on SSC-Area and FSC-Area for morphology to remove debris. PI and Annexin V or PI and Hoechst for apoptosis assays.

- [x] Tick this box to confirm that a figure exemplifying the gating strategy is provided in the Supplementary Information.
