# Supplementary material for: CRISPR screens reveal convergent targeting strategies against evolutionarily distinct chemoresistance in cancer
Source: Nat Commun. 2024 Jun 29;15:5502. doi: 10.1038/s41467-024-49673-4 (PMC11217446; doi:10.1038/s41467-024-49673-4)
Supplement: Supplementary file 9 — Description of Additional Supplementary Files [file 41467_2024_49673_MOESM9_ESM.pdf]

Description of Additional Supplementary Files

File Name: Supplementary Data 1
Description: First-round genome-scale CRISPR screen data.

File Name: Supplementary Data 2
Description: RNA-seq data for chemoresistant cells.

File Name: Supplementary Data 3
Description: Druggable gene library for CRISPR knockout screening.

File Name: Supplementary Data 4
Description: Second-round druggable gene library CRISPR screen data.

File Name: Supplementary Data 5
Description: RNA-seq data for PLK4 target genes.

File Name: Supplementary Data 6
Description: Oligonucleotides and primer sequences.
